# A Rabies Virus Glycoprotein Subunit Vaccine Produced in *Pichia pastoris* Induces Neutralizing Antibodies in Mice

**DOI:** 10.3390/vaccines14040322

**Published:** 2026-04-04

**Authors:** Ye Yang, Ruo Mo, Zhuoran Hou, Han Wang, Peng Sun, Ruixi Liu, Tiantian Wang, Bin Zhang, Xuchen Hou, Yongkun Zhao, Jun Wu, Bo Liu

**Affiliations:** 1 School of Life Sciences and Medical Enginering, Anhui University, Hefei 230601, China; yangye012023@163.com; 2 National Key Laboratory of Advanced Biotechnology, Academy of Military Medical Sciences, Beijing 100071, China; 3 Changchun Veterinary Research Institute, Chinese Academy of Agricultural Sciences, State Key Laboratory of Pathogen and Biosecurity, Key Laboratory of Jilin Province for Zoonosis Prevention and Control, Changchun 130000, China; 4 Tianjin Key Laboratory of Agricultural Animal Breeding and Healthy Husbandry, College of Animal Science and Veterinary Medicine, Tianjin Agricultural University, Tianjin 300392, China

**Keywords:** rabies virus, glycoprotein protein, recombinant subunit vaccine, CVS-11, *Pichia pastoris*, nanoparticles

## Abstract

**Background**: Rabies is a highly fatal zoonotic disease that causes approximately 59,000 human deaths worldwide each year. Current inactivated rabies vaccines require multiple doses and are associated with high costs. The full-length rabies virus glycoprotein (RVG), a membrane protein, exhibits substantial instability in its trimeric structure during recombinant expression. This instability makes it difficult to obtain high-purity, correctly folded antigens. **Objectives**: This study focuses on the preparation of a full-length recombinant RVG subunit vaccine candidate expressed in a glycoengineered *Pichia pastoris* system with mammalian-like glycosylation. **Methods**: The full-length RVG gene (including the transmembrane domain and cytoplasmic tail) from the Challenge Virus Standard-11 (CVS-11) strain was codon-optimized and inserted into the pPICZαA vector to construct the recombinant expression plasmid pPICZαA-RVG. The plasmid was transformed into glycoengineered *Pichia pastoris* X33-7 (low-mannose type) by electroporation for inducible expression. The target protein was purified by nickel affinity chromatography, anion-exchange chromatography, and Superdex-200 size-exclusion chromatography. The structural characteristics of the purified protein were analyzed by dynamic light scattering (DLS) and transmission electron microscopy (TEM). The purified antigen was formulated with the adjuvants AS03 or MF59. BALB/c mice (*n* = 5 per group) were immunized intramuscularly following a four-dose schedule (days 0, 7, 14, and 28). Antigen-specific IgG antibody titers were measured by ELISA, and neutralizing antibody titers were determined using the rapid fluorescent focus inhibition test (RFFIT). **Results**: Glycoengineered *Pichia pastoris* yeast strains expressing wild-type RVG (RVG-WT) or a mutant variant (RVG-M6: R84S, R199S, H270P, R279S, K300S, and R463S) were successfully constructed. The purified RVG antigen formed nanoparticles with an average particle size of approximately 75 nm. Immunized mice generated robust RVG-specific IgG responses, with titers reaching approximately 6.31 × 10^5^ for RVG-WT after the fourth immunization, compared to 3.16 × 10^3^ for RVG-M6 and 5.62 × 10^3^ for the RVG-WT-PEG control. Two weeks after the fourth immunization, RVG-WT formulated with AS03 or MF59 induced significant neutralizing antibody responses compared with the control group (*p* < 0.0001 and *p* < 0.01, respectively). The neutralizing antibody titers reached 1:79.43 in the AS03 group and 1:33.11 in the MF59 group, whereas the WT-PEG + AS03 control group showed a low titer of 1:3.72. In contrast, RVG-M6 formulated with MF59 failed to induce detectable neutralizing antibodies (1:3.02). Furthermore, RVG-WT + AS03 induced significantly higher neutralizing antibody responses than the WT-PEG + AS03 control group (*p* < 0.0001), and a significant difference was also observed between the RVG-WT + MF59 and RVG-M6 + MF59 groups (*p* < 0.01). **Conclusions**: The glycoengineered *Pichia pastoris* expression system successfully produced uniform full-length rabies virus glycoprotein nanoparticles with high purity. When formulated with the AS03 adjuvant, RVG-WT induced high-titer neutralizing antibodies in mice, suggesting a promising strategy for the development of recombinant subunit vaccines against rabies. However, this study is limited by the absence of challenge studies and validation in target animal species, which will be further investigated in future work.

## 1. Introduction

Rabies is an acute and fatal infectious disease of the central nervous system caused by the rabies virus (RABV), a member of the genus *Lyssavirus* in the family *Rhabdoviridae*. The rabies virus glycoprotein (RVG) is a transmembrane protein composed of an ectodomain, a transmembrane domain, and a cytoplasmic domain. The rabies virus genome encodes five structural proteins: nucleoprotein (N), phosphoprotein (P), matrix protein (M), glycoprotein (G), and RNA-dependent RNA polymerase (L). The G gene encodes a precursor protein consisting of 524 amino acids. This precursor is subsequently processed into a mature protein of 505 amino acids with a theoretical molecular weight of approximately 66 kDa [1].

The G protein is the only membrane protein located on the viral surface. It mediates viral attachment to host cell receptors and membrane fusion and serves as the primary antigen responsible for inducing protective neutralizing antibodies. Therefore, it represents the most important antigenic target for the development of recombinant subunit vaccines [2,3,4]. Several cellular receptors have been reported to mediate RABV entry, including the nicotinic acetylcholine receptor (nAChR), neural cell adhesion molecule (NCAM), p75 neurotrophin receptor (p75NTR), and metabotropic glutamate receptor 2 (mGluR2). These receptors facilitate viral internalization and membrane fusion [5,6,7,8]. RABV enters host cells through receptor-mediated endocytosis. Within the acidic environment of the endosome, the G protein undergoes conformational rearrangement. This rearrangement exposes fusion peptides, which then promote fusion between the viral envelope and the endosomal membrane. This process releases the viral ribonucleoprotein complex into the cytoplasm, thereby initiating viral replication and transcription [9,10].

Currently, commercially available rabies vaccines are primarily inactivated vaccines produced using cell culture systems, such as those derived from African green monkey kidney cells or human diploid cells [11,12,13,14]. These vaccines are widely administered for both pre-exposure prophylaxis (PrEP) in high-risk populations and post-exposure prophylaxis (PEP). However, most traditional rabies vaccines, which rely on completely inactivated viruses, are associated with several limitations. These include complex immunization schedules and relatively high production costs [15,16].

The glycoengineered *Pichia pastoris* expression system has garnered increasing attention in recombinant protein vaccine development due to its advantages, such as low cultivation costs, high expression levels, and the ability to perform eukaryotic glycosylation modifications [17,18,19,20]. Compared with prokaryotic expression systems, glycoengineered *P. pastoris* can produce proteins with glycosylation patterns that are more similar to those of mammalian cells, thereby reducing the risk of decreased immunogenicity caused by abnormal glycosylation [21,22]. To date, no recombinant subunit vaccine based on the rabies virus G protein has been successfully commercialized worldwide. The major technical challenges include the instability of membrane protein expression and the difficulty of obtaining high-purity target proteins through purification [3,23,24].

In this study, stable expression of the full-length RVG protein was achieved in glycoengineered *Pichia pastoris* through codon optimization, vector construction, and subsequent optimization of expression conditions. A three-step purification strategy was developed to obtain high-purity RVG nanoparticles. The immunogenicity and neutralizing antibody responses of the purified antigen were evaluated in BALB/c mice. These findings provide important technical support for the development of novel recombinant rabies subunit vaccines.

## 2. Materials and Methods

### 2.1. Glycoengineered Pichia pastoris X33-7, Bacterial Strains, and Materials

*Escherichia coli* Trans5α (TransGen Biotech, Beijing, China) was cultured in Luria–Bertani (LB) medium at 37 °C. The glycoengineered *Pichia pastoris* X33-7 strain used in this study was previously constructed and is routinely maintained in our laboratory under controlled conditions [25]. The X33-7 [20,25] strain was propagated in yeast extract–peptone–dextrose (YPD) medium at 25 °C. Yeast extract and tryptone used in the culture media were purchased from Oxoid (Basingstoke, UK). Zeocin was obtained from Thermo Fisher Scientific (Waltham, MA, USA).

### 2.2. Construction of Wild-Type RVG-WT and Mutant RVG-M6 Glycoprotein Expression Vectors and Screening of Positive Clones

The glycoprotein (RVG) gene from the CVS-11 strain was preserved in our laboratory (GenBank accession number: AAG34724.1). The RVG gene retains both the transmembrane domain (TM) and the cytoplasmic domain (CD). A flexible C-terminal linker (G_4_S) was introduced to connect the RVG antigen to the His-tag. The RVG-WT gene was cloned into the pPICZαA vector using *Xho* I and *Sac*I restriction sites to generate the pPICZαA-RVG-WT plasmid. For the mutant construct, the signal peptide of the RVG gene was removed, and six amino acid substitutions (R84S, R199S, H270P, R279S, K300S, and R463S) were introduced to generate the RVG-M6 variant [26]. The primer sequences used for RVG-M6 are listed in Supplementary Table S1. The modified gene was ligated into the pPICZαA vector using the *Xho* I and *Sac* I restriction sites, yielding the recombinant plasmid pPICZαA-RVG-M6. The linearized plasmids were subsequently transformed into the glycoengineered *Pichia pastoris* strain. Following methanol induction of protein expression in yeast cultures, positive clones were screened by Western blot analysis using an HRP-conjugated anti-His antibody (Sigma-Aldrich, Steinheim, Germany) at a 1:2000 dilution. Signals were detected using a chemiluminescence imaging system (BG-gdsAUTO710MINI, Beijing Baygene Biotechnology Co., Beijing, China).

### 2.3. Protein Purification of RVG-WT and RVG-M6

RVG-M6-positive clones identified by Western blot were further expanded in shaking flasks using a thermostatic incubator shaker (ZHWY-2112B, Shanghai Zhicheng Analytical Instrument Co., Shanghai, China), whereas RVG-WT-positive clones were cultivated in a 5 L bioreactor (BIOSTAT BPLUS, Sartorius, Göttingen, Germany). After yeast cultivation followed by methanol induction for 48 h, the cultures were centrifuged at 8000 rpm for 20 min to collect the cell pellets (J-26XP, Beckman Coulter, Brea, CA, USA). The pellets were resuspended in buffer and lysed to release intracellular proteins. Polyethylene glycol (PEG20000; Solarbio, Beijing, China) was added to the supernatant, followed by centrifugation to collect the precipitated pellet. The resulting pellet was dissolved in buffer and designated as the RVG-WT-PEG sample, which served as the control. The remaining samples were subjected to further purification. The RVG-WT protein was first purified by Ni Sepharose 6 Fast Flow affinity chromatography (Cytiva, New York, NY, USA) with gradient elution. The buffer was prepared at pH 7.5 with a salt concentration of 0.5 M NaCl. The eluted fractions were dialyzed and further purified by anion-exchange chromatography using Source 30Q resin (Cytiva, USA). The buffer was prepared at pH 8.0 with a salt concentration of 20 mM~1 M NaCl. The sample was subsequently concentrated by ultrafiltration (UF) (Merk, Darmstadt, Germany) and separated using a Superdex-200 size-exclusion (Cytiva, USA) chromatography column to obtain the purified protein (buffer: 1XPBS + 0.1% Tween-80). The purified products were analyzed by 10% SDS-PAGE using an electrophoresis system (DYY-6C, Beijing Liuyi Biotechnology Co., Beijing, China). Proteins were transferred onto membranes using an eBlot™ L1 rapid transfer system (GenScript, Nanjing, China). In summary, the target protein was obtained through a two-step purification strategy consisting of Ni-affinity chromatography followed by Superdex-200 size-exclusion chromatography. For Western blot analysis of RVG-WT, the membranes were sequentially incubated with a rabies virus polyclonal antibody (1:2000) as the primary antibody and a goat anti-rabbit HRP-conjugated secondary antibody (1:5000) (Yiqiao Shenzhou, Beijing, China). Purification of RVG-M6 was verified by Western blot using an HRP-conjugated anti-His antibody (1:2000) (Sigma-Aldrich, Germany).

### 2.4. Structural Features and Characterization of RVG-WT and RVG-M6 Proteins

The purified RVG-WT and RVG-M6 protein preparations were treated with peptide-N-glycosidase F (PNGase F; stored in our laboratory) to remove N-linked glycans. The reaction mixtures were incubated overnight at 37 °C in a constant-temperature incubator. The treated samples were subsequently analyzed by 12% SDS-PAGE and Western blot. For Western blot analysis, RVG-WT and RVG-M6 were probed with a rabies virus polyclonal antibody (1:2000) as the primary antibody, followed by a goat anti-rabbit HRP-conjugated secondary antibody (1:5000) (Yiqiao Shenzhou, Beijing, China). The purified RVG-WT product obtained through molecular sieve purification was analyzed using a particle size analyzer (Zetasizer Advance, Malvern Panalytical, Beijing, China) to determine the particle size and distribution of the protein nanoparticles.

### 2.5. Vaccine Preparation and Animal Immunization

Five groups of 6–8-week-old female BALB/C mice (five per group) were purchased from Beijing Vital River Laboratory Animal Technology Co., Ltd. (Beijing, China) and maintained in a specific pathogen-free (SPF) animal facility. After a one-week acclimatization period, the mice were used for subsequent experiments. The sample size (*n* = 5 per group) was determined with reference to previously published studies [27].

All animal experiments were approved by the Institutional Animal Care and Use Committee (Ethics No.: IACUC-2024-042).

For vaccine preparation, purified RVG-WT protein was formulated with either AS03 (Jiangsu Rec-Biotechnology Co., Ltd., Taizhou, China) or MF59 (AVT Pharmaceutical Tech Co., Ltd., Shanghai, China) adjuvant, while RVG-M6 protein was formulated with MF59 adjuvant. The purified proteins and adjuvants were thoroughly mixed at a 1:1 (*v*/*v*) ratio prior to immunization. In addition, the RVG-WT-PEG preparation formulated with AS03 adjuvant and physiological saline (Shandong Qidu Pharmaceutical Co., Ltd., Zibo, China) was included as the control group.

BALB/c mice were randomly assigned to experimental groups using a randomization procedure to minimize selection bias. The detailed immunization groups are listed in Table 1. Immunization was administered via intramuscular injection at a dose of 100 μL per mouse on days 0, 7, 14, and 28, with the schedule designed with reference to the post-exposure (PEP) immunization regimens commonly used for inactivated rabies vaccines [28,29]. We monitored and recorded the body weight and body temperature of the mice for one week following vaccination. The immunization dose was selected based on commonly used doses reported in the literature for similar vaccine candidates, as well as preliminary optimization experiments to ensure adequate immunogenicity.

### 2.6. Enzyme-Linked Immunosorbent Assay (ELISA)

Rabies virus antigen (Shandong Landu Biotechnology Co., Ltd., Jinan, China) was diluted to 2 μg/mL in 50 mmol/L carbonate coating buffer (pH 9.6). A volume of 100 μL of the diluted antigen was added to each well of a 96-well ELISA plate and incubated overnight at 4 °C for coating.

The following day, the plates were washed three times with PBST (phosphate-buffered saline containing 0.1% Tween-80). Each well was then blocked with 300 μL of 5% nonfat milk and incubated at 37 °C for 1 h. Serum samples were added in serial dilutions at a volume of 100 μL per well and incubated at 37 °C for 1 h. After incubation, the plates were washed four times with PBST.

Subsequently, 100 μL of HRP-conjugated goat anti-mouse IgG (Yiqiao Shenzhou, Beijing, China) was added to each well and incubated at 37 °C for 1 h, followed by four washes with PBST. Then, 100 μL of TMB one-component chromogenic substrate (Solarbio, PR1200) was added to each well and incubated for 5 min at room temperature. The reaction was terminated by adding 50 μL of stop solution (Solarbio, Beijing, China). The absorbance was measured at 450 nm using a microplate reader (Spectra Max, Molecular Devices, San Jose, CA, USA).

### 2.7. Viral Neutralization Test (RFFIT)

Virus neutralization tests were performed using the ERA strain expressing enhanced green fluorescent protein (EGFP). Fifty microliters of each sample (serum from individual mice within the same group) was used as the positive reference control (0.5 IU/mL). The standard serum used for FAVN testing was obtained from the EU Rabies Reference Laboratory in France and the negative control was serum obtained from the Normal saline group.; these were added into four consecutive wells of a 96-well plate and subjected to a serial threefold dilution. An equal volume (50 μL) of RABV-ERA-EGFP containing 100 TCID_50_/50 μL was added to each dilution and incubated for 1 h at 37 °C to facilitate antibody–virus interaction. Following incubation, BHK-21 cell suspensions were added to each well and maintained under standard culture conditions for 72 h. The assay was performed in accordance with WOAH guidelines with minor laboratory-specific optimization of incubation time. Plates were examined under a fluorescence microscope, and the neutralizing antibody titers were calculated in IU/mL by comparison with the reference standard, as described previously [30].

### 2.8. Statistical Analysis

Data processing and visualization were performed using GraphPad Prism 9.0.0 software. For analyses involving multiple parallel groups, one-way analysis of variance (ANOVA) was conducted, followed by multiple comparison tests. Statistical significance criteria were as follows: *p* > 0.05, ns (not significant); *p* < 0.05 (*), *p* < 0.01 (**), *p* < 0.001 (***), *p* < 0.0001 (****). All results are expressed as mean ± standard deviation (mean ± SD).

## 3. Results

### 3.1. Expression and Purification of RVG-WT and RVG-M6 in the Glycoengineered Pichia pastoris

**Figure 1:**
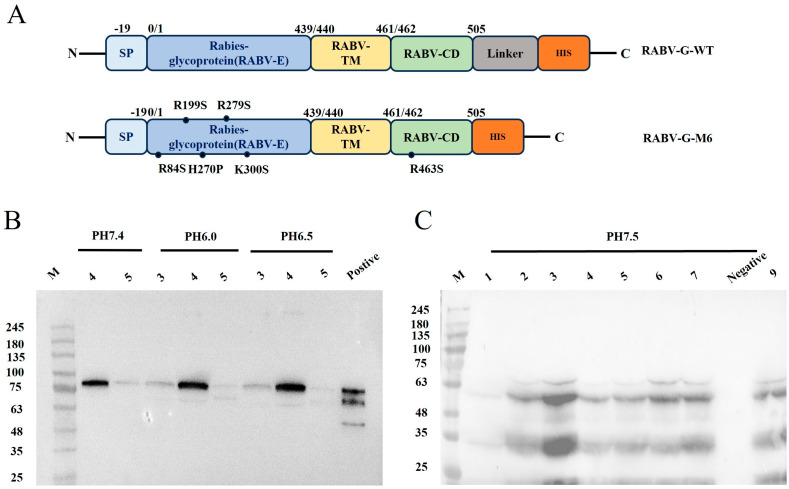
Antigen Preparation and Screening of RVG-WT and RVG-M6: (**A**) Gene maps of RVG-WT and RVG-M6. (**B**) Western blot results of positive clone screening for RVG-WT induced under three pH conditions in yeast; 3, 4, and 5 represent clone numbers for screening. (**C**) Western blot results of positive clone screening for RVG-M6 induced at pH 7.5. In this image, 1–7 and 9 represent clone numbers for screening. The original Western blot figures can be found in Supplementary Figure S1.

**Figure 2:**
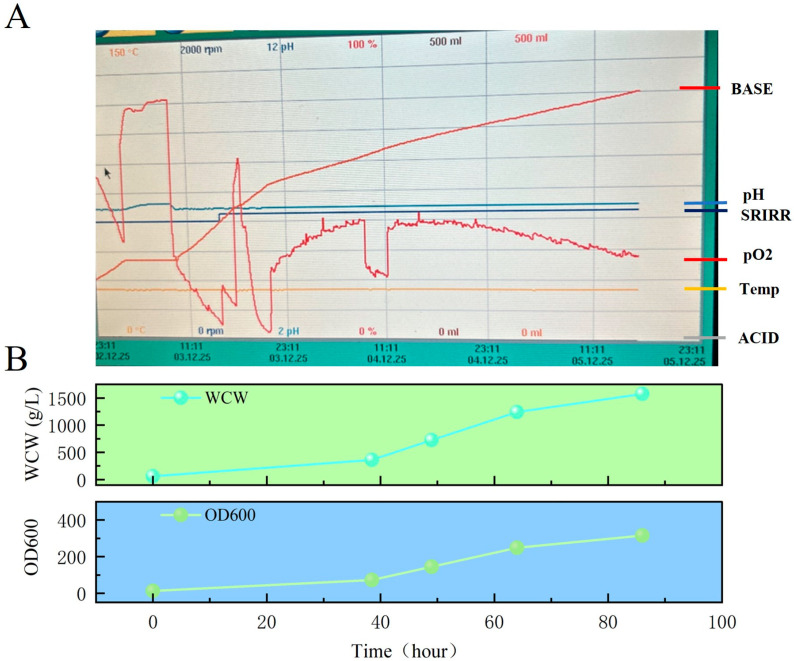
Growth curves of wet cell weight and yeast cell density during RVG-WT fermentation: (**A**) RVG-WT fermentation process curve. (**B**) RVG-WT fermentation process curves: cell weight (**upper panel**) and cell density (**lower panel**).

Positive clones of RVG-WT and RVG-M6 identified by Western blot (Figure 1B,C) were selected for scale-up and protein expression. RVG-M6 was cultured in shake flasks, whereas RVG-WT was produced using bioreactor fermentation (Figure 2A).

**Figure 3:**
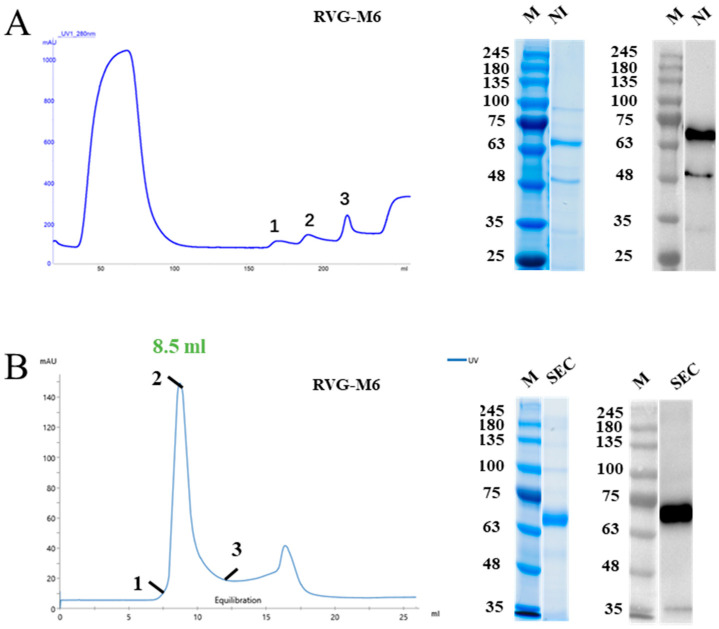
Chromatogram of purified RVG-M6 antigen genes along with corresponding SDS-PAGE and Western blot results: (**A**) RVG-M6 Ni-affinity chromatography purification profile, along with SDS-PAGE and Western blot analyses of the eluate from position 2. (**B**) RVG-M6 Superdex-200 purification profile, along with SDS-PAGE and Western blot analyses of the eluate from position 2. The original Western blot figures and SDS-PAGE figures can be found in Supplementary Figure S1.

For RVG-M6 expression, positive clone #3 was selected for shake-flask fermentation. After initial purification using Ni-affinity chromatography, SDS-PAGE and Western blot analyses revealed a protein band at approximately 66 kDa, which was consistent with the predicted molecular weight (Figure 3A). The Ni-purified RVG-M6 protein was further subjected to size-exclusion chromatography using a Superdex-200 column (Figure 3B). The chromatographic profile indicated that the target protein eluted at approximately 8.5 mL, and the corresponding SDS-PAGE and Western blot results demonstrated improved purity of the eluted fractions (Figure 3B).

**Figure 4:**
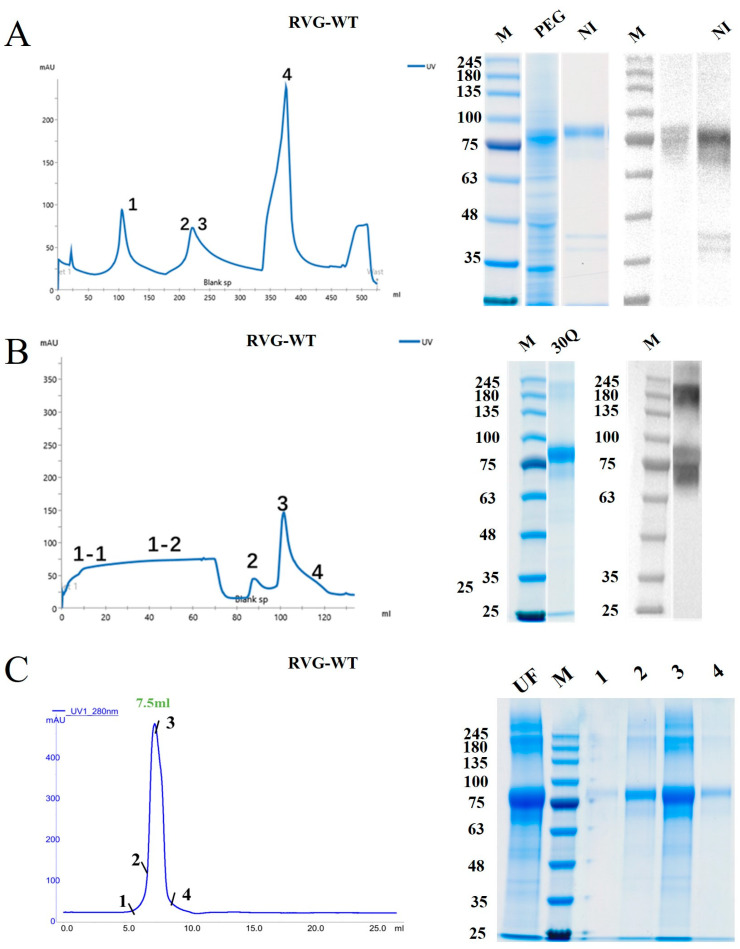
Chromatogram of the purified RVG-WT antigen gene, along with corresponding SDS-PAGE and Western blot results: (**A**) Ni-affinity chromatography purification profile of RVG-WT, along with SDS-PAGE and Western blot analyses of the PEG-treated sample and Ni-affinity-eluted purified sample 4. (**B**) RVG-WT Source-30Q purification profile and corresponding SDS-PAGE and Western blot analyses of sample 1-1. (**C**) Superdex-200 purified chromatogram of RVG-WT and the corresponding SDS-PAGE analysis of the sample. The original Western blot figures and SDS-PAGE figures can be found in Supplementary Figure S1.

For RVG-WT expression, induction was evaluated under three different pH conditions. Among the tested clones, clone #4 induced at pH 6.5 showed the most suitable expression characteristics and was selected for subsequent bioreactor fermentation (Figure 1B). During fermentation, both biomass and cell density increased progressively over time (Figure 2B). The first-step purification of RVG-WT using Ni-affinity chromatography yielded gradient-eluted fractions, and the SDS-PAGE and Western blot results for RVG-WT-PEG and RVG-WT are shown in Figure 4A. The RVG-WT protein exhibited an apparent molecular weight of approximately 80 kDa. Subsequent purification using an anion-exchange chromatography column further increased the purity of the protein (Figure 4B).

Finally, purification was achieved by Superdex-200 size-exclusion chromatography. The chromatogram showed that the target protein eluted as a single peak at approximately 7.5 mL [3]. SDS-PAGE analysis confirmed the high purity of the collected fractions (Figure 4C).

### 3.2. Glycosylation Characterization and Particle Size Analysis of Purified RVG-WT and RVG-M6

**Figure 5:**
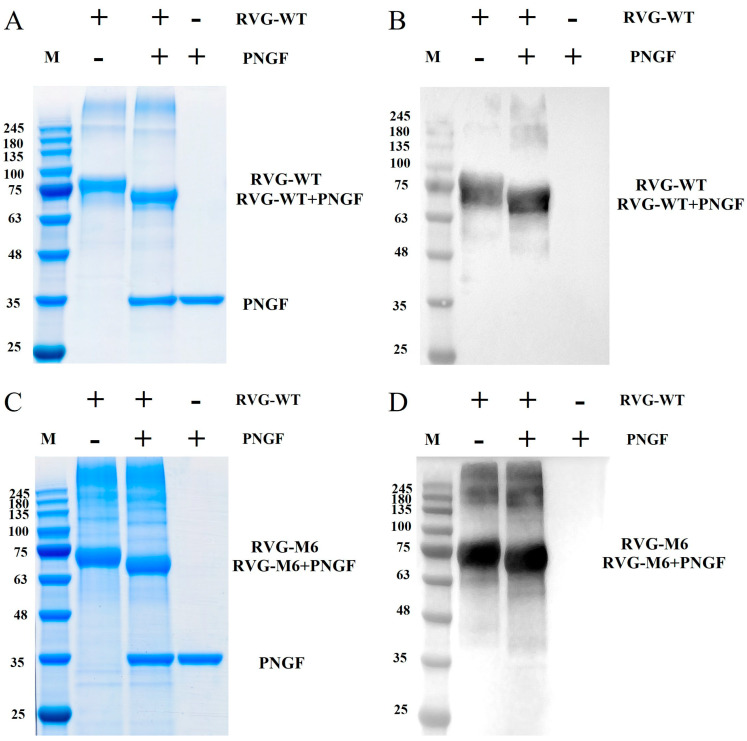
N-glycosylation identification of RVG-WT and RVG-M6: (**A**) SDS-PAGE result for N-glycosylation identification of RVG-WT protein. (**B**) Western blot (**right**) result for N-glycosylation identification of RVG-WT protein. (**C**) SDS-PAGE result for N-glycosylation identification of RVG-M6 protein. (**D**) Western blot (**right**) result for N-glycosylation identification of RVG-M6 protein. “+” indicates the addition of antigen protein or PNGase F enzyme, while “−” indicates no addition of antigen protein or PNGase F enzyme. The original Western blot figures and SDS-PAGE figures can be found in Supplementary Figure S1.

Both RVG-WT and RVG-M6 proteins contain four potential N-glycosylation sites. Following digestion with PNGase F, SDS-PAGE analysis showed that the molecular weights of RVG-WT (Figure 5A) and RVG-M6 (Figure 5C) expressed in glycoengineered *Pichia pastoris* shifted to values consistent with their predicted molecular weights. Western blot analysis further revealed a clear shift of the target bands after deglycosylation, confirming the presence of N-linked glycosylation modifications (Figure 5B,D).

**Figure 6:**
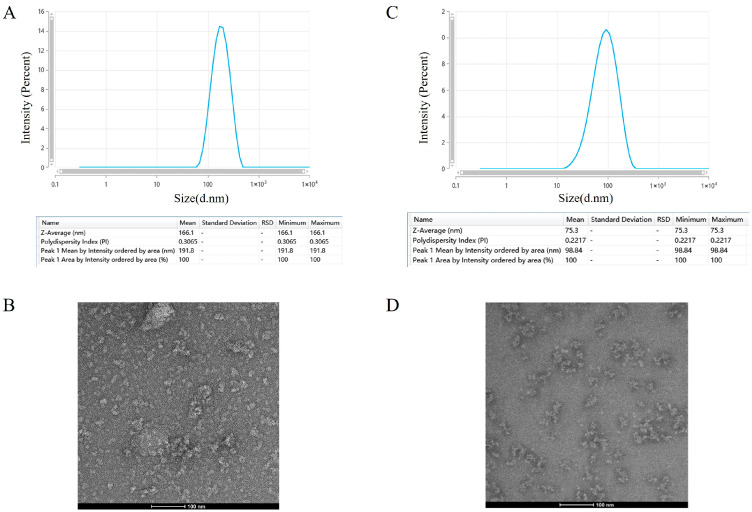
Structural characterization of RVG-WT and RVG-M6 under microscopic conditions: (**A**) Uniformity of nanoparticle size and distribution of RVG-M6 under particle size analysis. (**B**) Nanoparticle distribution of RVG-M6 under transmission electron microscopy. (**C**) Structural characterization of RVG-WT and RVG-WT under microscopic conditions. (**D**) Nanoparticle distribution of RVG-WT under transmission electron microscopy.

Particle size analysis was subsequently performed to characterize the structural properties of the purified proteins. The RVG-WT particles exhibited an average diameter of approximately 75 nm, with a polydispersity index (PDI) of 0.22, indicating relatively uniform particle distribution (Figure 6C). In contrast, RVG-M6 particles displayed a significantly increased average diameter (~166 nm) and a higher PDI (0.31), indicating a less uniform size distribution and the possible presence of larger particle species compared to RVG-WT (Figure 6A).

Transmission electron microscopy (TEM) confirmed that RVG-WT formed uniformly distributed nanoparticle structures (Figure 6D). In contrast, TEM analysis of RVG-M6 revealed a heterogeneous particle distribution with evident aggregation (Figure 6B), which is consistent with the increased particle size and higher PDI observed in the particle size analysis. These results suggest that the RVG-WT protein expressed in glycoengineered *Pichia pastoris* can self-assemble into nanoparticle-like structures after purification.

### 3.3. High Titers of Specific IgG Antibodies Were Induced in Mice After Four Immunizations

**Figure 7:**
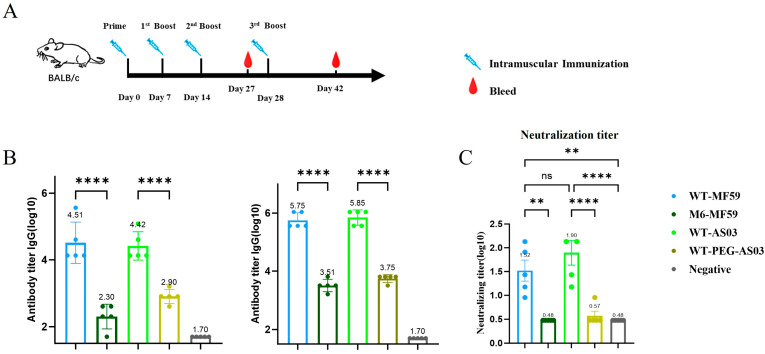
Immunization schedule and post-vaccination antibody titers and neutralizing antibody levels in BALB/c mice immunized with RVG-WT and RVG-M6 recombinant subunit vaccines: (**A**) Experimental timeline showing vaccination (blue syringe) and serum collection (red droplet) within 6 weeks. Mice were immunized via intramuscular injection, with groups including normal saline, RVG-WT-PEG + AS03, RVG-WT + AS03, RVG-WT + MF59, and RVG-M6 + MF59. (**B**) Anti-RVG IgG levels in serum at days 27 (**left**) and 42 (**right**). (**C**) Neutralizing capacity against the ERA virus in immunized mice using different adjuvants and different antigens (*n* = 5). ns, not significant; ** *p* < 0.01; **** *p* < 0.0001.

To evaluate the immunogenicity of the G protein-based recombinant subunit vaccine, mice were immunized according to a four-dose schedule on days 0, 7, 14, and 28 (Figure 7A). The purified RVG-WT protein was formulated with either AS03 or MF59 adjuvants, while the RVG-WT-PEG control preparation was formulated with the AS03 adjuvant (Table 1). Saline was used as the negative control. BALB/c mice were immunized via intramuscular injection.

Serum samples were collected 2 weeks after the third and fourth immunizations, and specific IgG antibody levels were determined by ELISA (Figure 7B). Mice immunized with purified RVG-WT formulated with either MF59 or AS03 adjuvants produced high levels of specific IgG antibodies. Two weeks after the fourth immunization, the IgG antibody titer in mice immunized with RVG-WT reached approximately 6.31 × 10^5^. In contrast, mice immunized with RVG-M6 formulated with MF59 generated a significantly lower antibody titer of approximately 3.16 × 10^3^ after the fourth immunization. Similarly, the RVG-WT-PEG control formulated with AS03 induced an IgG titer of approximately 5.62 × 10^3^.

Statistical analysis showed that the antibody titers induced by the RVG-WT + AS03 group were significantly higher than those induced by the RVG-WT-PEG + AS03 control group (*p* < 0.0001). In addition, when formulated with MF59 adjuvant, the antibody response induced by RVG-WT was significantly higher than that induced by RVG-M6 (*p* < 0.0001). These results indicate that the purified RVG-WT exhibits substantially stronger immunogenicity compared with RVG-M6 and RVG-WT-PEG.

### 3.4. Neutralizing Antibody Responses Induced After the Fourth Immunization

Neutralizing antibody responses induced by the vaccines were evaluated two weeks after the fourth immunization using a rapid fluorescent focus inhibition test (RFFIT). The results showed that mice immunized with RVG-WT formulated with either AS03 or MF59 adjuvants produced detectable rabies virus-neutralizing antibodies (Figure 7C).

Compared with the negative control group, the RVG-WT + MF59 group generated significantly higher neutralizing antibody titers (*p* < 0.01), reaching a mean titer of 1:33.11. In addition, the neutralizing antibody levels in the RVG-WT + MF59 group were significantly higher than those in the RVG-M6 + MF59 group (*p* < 0.01).

Similarly, mice immunized with RVG-WT + AS03 produced strong neutralizing antibody responses, with titers reaching 1:79.43, which were significantly higher than those in the negative control group (*p* < 0.0001) and the RVG-WT-PEG + AS03 control group (*p* < 0.0001). In contrast, the RVG-WT-PEG + AS03 group showed minimal viral neutralizing activity, with a low titer of 1:3.72, comparable to the normal saline group.

Notably, RVG-M6 formulated with MF59 failed to induce detectable neutralizing antibodies, with titers remaining at a baseline level (1:3.02), suggesting that the introduced mutations may have disrupted critical neutralizing epitopes of the G protein. No significant difference in neutralizing antibody titers was observed between the RVG-WT + AS03 and RVG-WT + MF59 groups (*p* > 0.05). The detailed quantitative results are presented in Table 2.

### 3.5. Preliminary Assessment of Vaccine Safety

**Figure 8:**
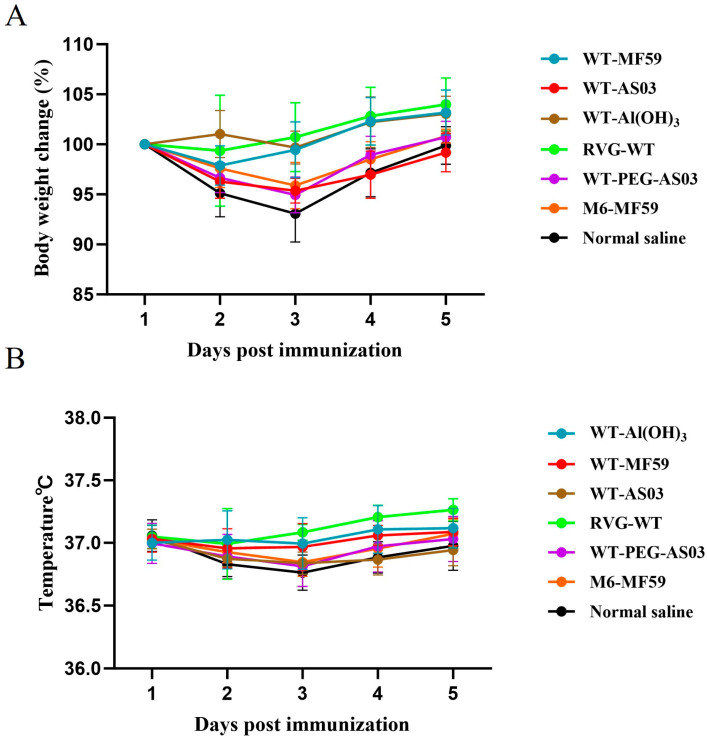
Evaluation of safety based on body weight and body temperature changes in mice within one week following immunization with RVG-WT and RVG-M6 formulations: (**A**) Body weight changes in mice during the first week post-immunization. (**B**) Body temperature changes in mice during the first week post-immunization (*n* = 5).

Within one week after immunization, the body weight and body temperature of mice were monitored to preliminarily evaluate the safety of the vaccine. As shown in the results, a slight decrease in body weight was observed on day 2 post-immunization, which was consistent with the trend observed in the normal saline control group. The body weight gradually recovered from day 3 onwards, and no significant or sustained weight loss was observed (Figure 8A). Body temperature monitoring showed that the temperature of mice fluctuated within the range of 36.0–37.5 °C, which is within the normal physiological range for mice. No abnormal temperature changes were detected during the observation period (Figure 8B).

These findings indicate that immunization with the candidate vaccine does not induce obvious systemic adverse effects in mice, as reflected by stable body weight and normal body temperature. Nevertheless, given that this is a preliminary safety assessment, additional studies are warranted to further evaluate potential immunological and pathological effects in both laboratory and target animal species.

## 4. Discussion

The high fatality rate of rabies highlights the urgent need for the development of novel and highly effective vaccines. Currently available rabies vaccines are primarily inactivated vaccines produced through cell culture and viral inactivation processes. Although these vaccines are effective, they are associated with several limitations, including high production costs and complex immunization schedules [15]. Recombinant subunit vaccines contain only purified antigenic proteins without viral genetic material, offering improved safety profiles. Moreover, they can be produced at a large scale using engineered expression systems, which may significantly reduce manufacturing costs.

As the only surface protein of the rabies virus, the rabies virus glycoprotein (RVG) is the primary target for virus-neutralizing antibodies. Therefore, the structural integrity of RVG directly determines the immunogenicity of rabies vaccines. However, wild-type full-length RVG is prone to degradation during recombinant expression and purification processes, which poses a major challenge for vaccine development [27,31,32]. In this study, two strategies were explored to address this issue. The first strategy involved inhibiting proteolytic degradation through targeted point mutations [26], while the second strategy utilized the addition of protease inhibitors during protein preparation. Importantly, both approaches retained the transmembrane domain (TM) and cytoplasmic domain (CD) of the protein to preserve the integrity of neutralizing antibody epitopes [33,34].

The glycoengineered *Pichia pastoris* expression system has been genetically modified to perform mammalian-like glycosylation, which helps maintain the native conformation and immunogenicity of recombinant proteins [20,35,36]. In the present study, our findings support the conclusion reported by Michelle C. Crank et al. that substitutions of basic amino acids can reduce protease susceptibility [26]. Although the RVG-M6 variant containing basic amino acid substitutions effectively reduced protein degradation, these mutations likely altered critical neutralizing epitopes, resulting in the absence of detectable neutralizing antibody responses in mice. Further structural characterization using techniques such as mass spectrometry and cryo-electron microscopy may help clarify the structural alterations caused by these mutations. The antigen in this study was produced using the *Pichia pastoris* expression system, which is widely regarded as a cost-effective platform due to its high yield, low culture cost, and scalability. Although a detailed cost analysis was not performed, this system is considered suitable for large-scale vaccine production [3,37]. Compared with mammalian cell expression systems, yeast-based expression platforms are more cost-effective and exhibit stronger market competitiveness [27,38].

In this study, stable expression of RVG was achieved in *Pichia pastoris* through codon optimization. PNGase F digestion confirmed the presence of N-linked glycosylation. Western blot analysis showed an apparent molecular weight of approximately 80 kDa, suggesting that the glycoengineered *Pichia pastoris* system successfully introduced appropriate N-glycosylation modifications to RVG [35,36]. Efficient purification of membrane proteins remains a major technical challenge in recombinant vaccine development. To address this, a three-step purification strategy was established, consisting of Ni-affinity chromatography, anion-exchange chromatography, and Superdex-200 size-exclusion chromatography. Ni-affinity chromatography enabled initial enrichment of the target protein through the C-terminal 6 × His tag. Subsequently, anion-exchange chromatography removed acidic impurities, while size-exclusion chromatography further separated highly uniform nanoparticle antigens based on molecular size and conformational differences. In future studies, we plan to further characterize the glycosylation of this antigen using mass spectrometry.

Particle size analysis and electron microscopy demonstrated that RVG-WT formed nanoparticles with an approximate diameter of 75 nm. Such nanoparticle structures allow multivalent presentation of antigenic epitopes, which can enhance antigen recognition and uptake by antigen-presenting cells. This multivalent display may contribute to the strong immune responses associated with protection observed in the immunized mice. In contrast, RVG-M6 exhibited a markedly increased particle size (~166 nm) with a higher polydispersity index, along with heterogeneous morphology and evident aggregation as observed by TEM. These structural alterations suggest that the mutations may impair the proper assembly and stability of the protein nanoparticles. Such aggregation and reduced uniformity could potentially hinder the optimal exposure of neutralizing epitopes and limit efficient interaction with antigen-presenting cells. Consequently, these structural differences may partly explain the comparatively weaker neutralizing antibody responses observed for RVG-M6.

The neutralizing efficacy of the recombinant rabies subunit vaccine was evaluated using the log_10_ virus neutralization titer as an indicator [1,39]. The results showed that the post-immunization serum neutralizing antibody titer was 1:79.43, which was significantly higher than that of the negative control group. These findings indicate that the recombinant RVG-WT antigen effectively induces rabies virus-specific neutralizing antibodies in mice. The RVG-WT antigen used in this study retains key neutralizing epitopes and preserves its native conformation, enabling efficient activation of humoral immune responses associated with protection. As a result, immune sera were able to inhibit viral infection even at relatively high dilution levels.

Further studies could explore strategies to reduce the number of immunizations required, such as optimizing immunization schedules or evaluating additional adjuvants. Increasing sample sizes and testing different adjuvant formulations may further improve the immunogenicity of RVG-WT. In contrast, the RVG-M6 variant failed to induce neutralizing antibodies, likely due to the disruption of key neutralizing epitopes caused by alkaline amino acid substitutions. Future studies may focus on identifying specific mutation sites that influence epitope integrity.

Although the immunogenicity and neutralizing activity of this vaccine candidate were demonstrated in mice, vaccine efficacy was primarily assessed based on neutralizing antibody responses, without validation through challenge studies or comprehensive evaluation of cellular immune responses, such as T-cell immunity. In addition, safety assessment was limited to preliminary observations of body weight and temperature. Therefore, further studies are required to evaluate the efficacy and safety of this vaccine in target animal models more relevant to clinical application, such as dogs, bats, and other wildlife species.

## 5. Conclusions

The glycoengineered *Pichia pastoris* system produced uniform full-length rabies virus G protein nanoparticles with high purity. When combined with the AS03 adjuvant, RVG-WT induced high-titer protective neutralizing antibodies in mice, providing a viable technical approach for developing recombinant subunit vaccines against rabies.

## Figures and Tables

**Table 1:** Animal immunization group information.

Vaccine Group	RVG-WT (μg/Dose)	RVG-WT-PEG (μg/Dose)	RVG-M6 (μg/Dose)	AS03 (*v*/*v*)	MF59 (*v*/*v*)
RVG-WT-AS03	10	/	/	1:1	/
RVG-WT-MF59	10	/	/	/	1:1
RVG-WT-PEG-AS03	/	20 (Equivalent product)	/	1:1	/
RVG-M6-MF59	/	/	10	/	1:1
Normal saline	/	/	/	/	/

**Table 2:** Neutralizing antibody titers (log_10_) in mice at 42 days post-immunization with different RVG-based vaccine formulations.

Group	Time After Immunization	Neutralization Titer (log10)	Geometric Mean (log10)
RVG-WT-AS03	42	2.13 1.18 1.43 2.61 2.13	1.90
RVG-WT-MF59	42	0.96 1.43 2.13 1.91 1.18	1.51
WT-PEG-AS03	42	0.48 0.48 0.48 0.48 0.48	0.48
RVG-M6-MF59	42	0.48 0.48 0.48 0.48 0.48	0.48
Normal saline	42	0.48 0.48 0.48 0.48 0.48	0.48
Positive	-	1.91 1.91 1.91 1.91	1.91

## Data Availability

The raw data supporting the conclusions of this article will be made available by the authors, without undue reservation.

## Supplementary Materials

The following supporting information can be downloaded at: https://www.mdpi.com/article/10.3390/vaccines14040322/s1, Figure S1: The original Western blot and SDS-PAGE figures; Table S1: The primer sequences used for RVG-M6.

## Abbreviations

The following abbreviations are used in this manuscript:

ANOVA	Analysis of variance
CD	Cytoplasmic domain
DLS	Dynamic light scattering
ECD	Extracellular domain
EGFP	Enhanced green fluorescent protein
PDI	Polydispersity index
PEP	Post-exposure prophylaxis
PrEP	Pre-exposure prophylaxis
SPF	Specific pathogen-free
TM	Transmembrane domain
ELISA	Enzyme-linked immunosorbent assay
RABV	Rabies virus
RVG	Rabies virus glycoprotein
SDS-PAGE	SDS–polyacrylamide gel electrophoresis
kDa	Kilodalton
