# Supplementary material for: A Rabies Virus Glycoprotein Subunit Vaccine Produced in Pichia pastoris Induces Neutralizing Antibodies in Mice
Source: Vaccines (Basel). 2026 Apr 4;14(4):322. doi: 10.3390/vaccines14040322 (PMC13119759; doi:10.3390/vaccines14040322)
Supplement: Supplementary file 1 [file vaccines-14-00322-s001.zip › vaccines-4215229-supplementary.pdf]

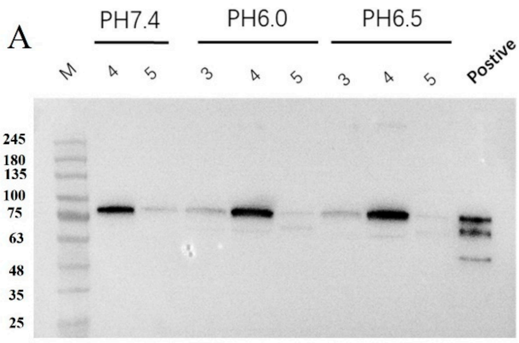

FIG 1A

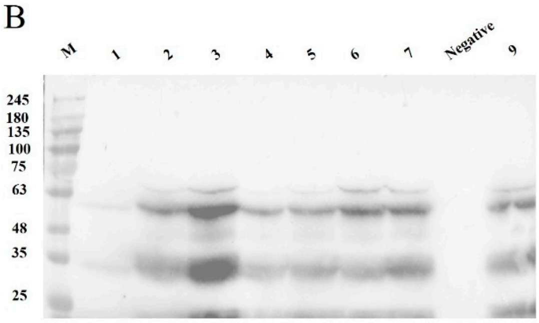

FIG 1B

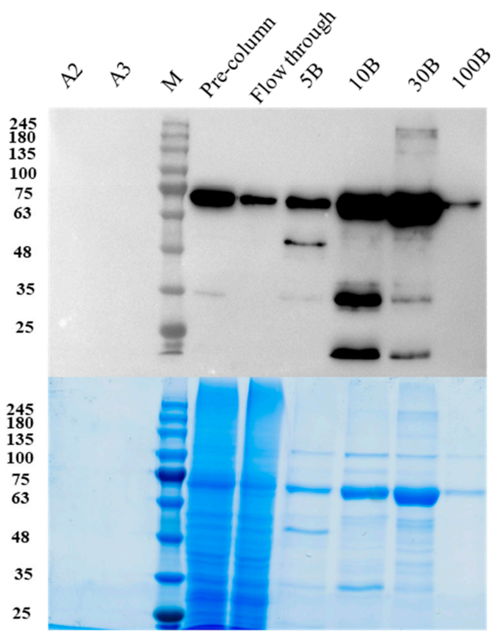

Fig3A

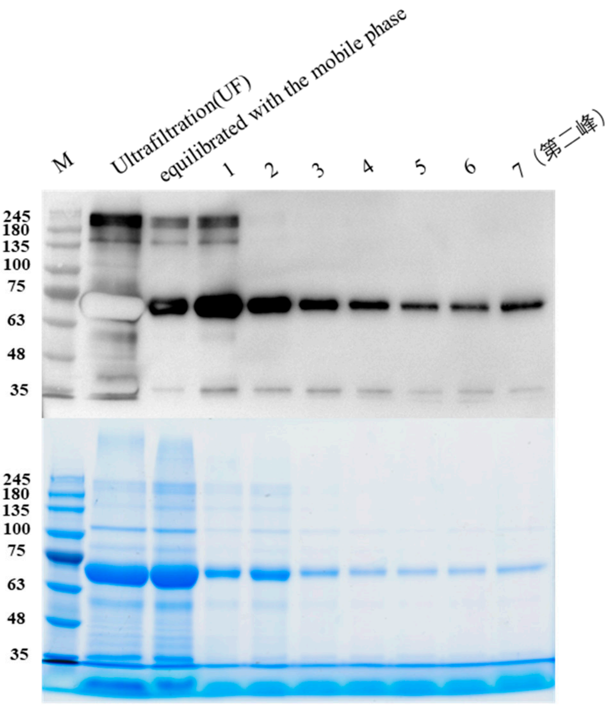

Fig3B

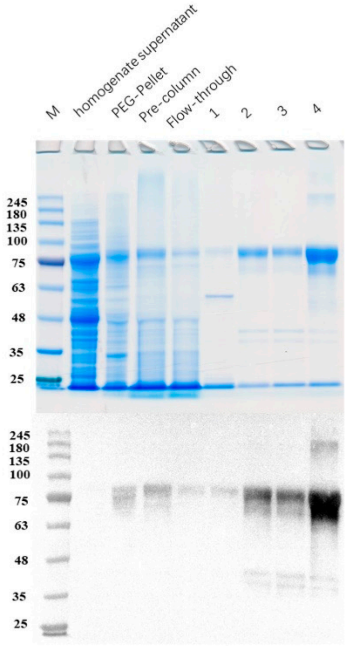

Fig4A

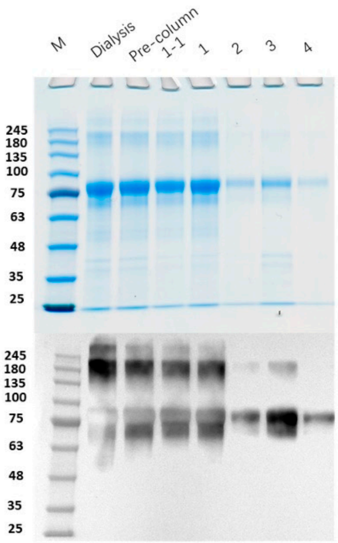

Fig4B

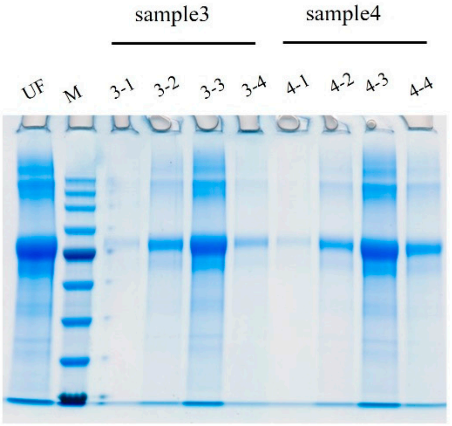

Fig4C

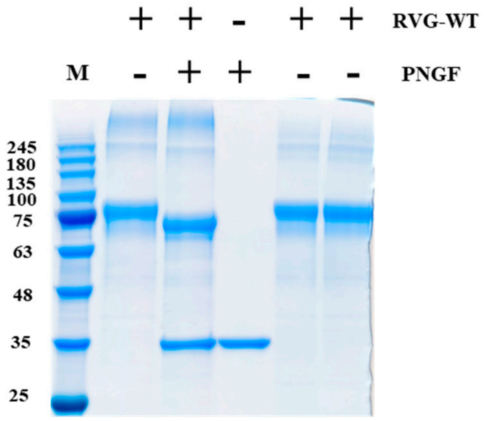

Fig5 A

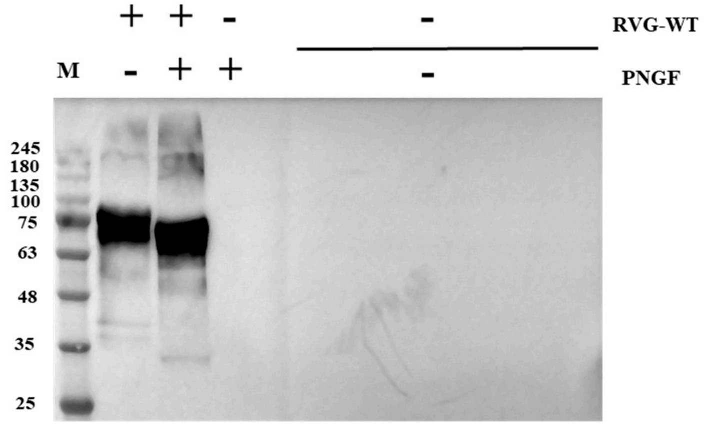

Fig5 B

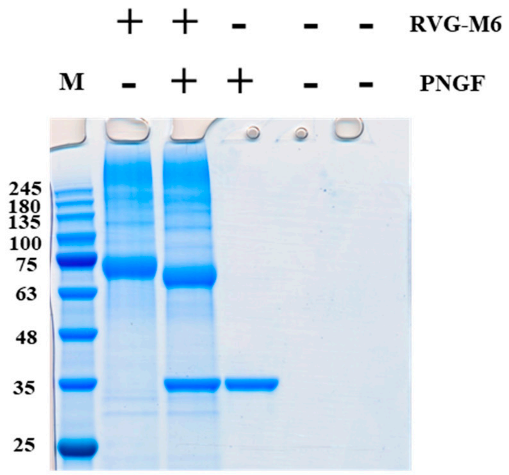

Fig5 C

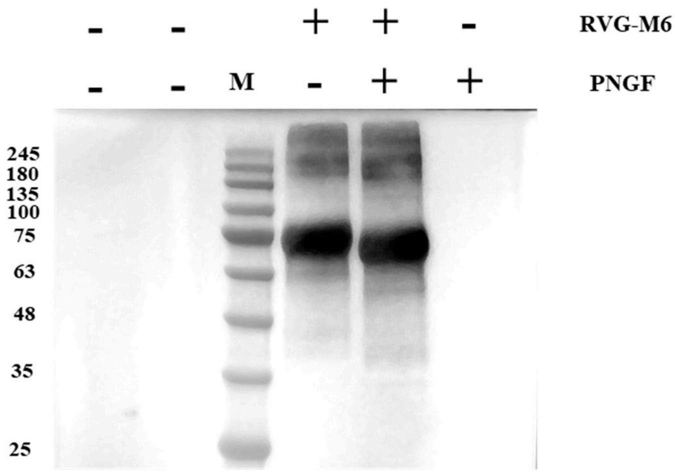

Fig5 D

Figure S1. The original Western blot and SDS-PAGE figures.

Table S1. The primer sequences used for RVG-M6.

Primer	Sequence (5′–3′)
RABV-G-5′	GAAGGGGTATCTCTCGAGAAAAGAAAATTCCCAATCTACACT
RV-G-3′	TCAATGATGATGATGATGATGGTCGACCAATTTAGTCTCTCCACC
R84S-5′	GTCACTACAACCTTTAAGCGAAAACACTTCAGACCAACTCCT
R84S-3′	GCTCTTAAAGGTTGTAGTGACATATCC
R199S-5′	ACCAACTCCAGAGGAAAGCGAGCTTCTAAGGGTTCCAAAACAT
R199S-3′	GCTCTTTCCTCTGGAGTTGGTAAAGAT
H270P+R279S-5′	CATTTGGTTGTCGAAGAGCTTGTTGAAAAGAGCGAAGAGTGTCTTGATGCTTTG
H270P+R279S-3′	TCTCTTTTCAACAAGCTCTTCGACAACCAAATGCTCAA
K306S-5′	AGACTTAGTCCTTTGAGAAGACTTGTCCCAGGTTTCGGAAAAG
K306S-3′	TCGTCTCAAAGGACTAAGTCTTCTGAATGAAACTGGGTTAGT
R463S-5′	TTCTTGATGACCTGTTGCAGAAGCACTAATAGAGCTGAATCTAT
R463S-3′	GCTTCTGCAACAGGTCATCAAGAAAAT
